# Rho‐associated protein kinase‐dependent moesin phosphorylation is required for PD‐L1 stabilization in breast cancer

**DOI:** 10.1002/1878-0261.12804

**Published:** 2020-10-03

**Authors:** Fanbiao Meng, Yang Su, Bo Xu

**Affiliations:** ^1^ Department of Biochemistry and Molecular Biology, Tianjin Medical University Cancer Institute and Hospital, Key Laboratory of Breast Cancer Prevention and Therapy, Ministry of Education National Clinical Research Center for Cancer, Key Laboratory of Cancer Prevention and Therapy, Tianjin, Tianjin’s Clinical Research Center for Cancer Tianjin China; ^2^ Center for Intelligent Oncology, Chongqing University Cancer Hospital, Chongqing University School of Medicine Chongqing China

**Keywords:** MSN, PD‐L1, ROCK inhibitor, T‐cell immune response

## Abstract

Expression of programmed cell death ligand (PD‐L1) is associated with poor prognosis in breast cancer. Understanding the regulation of PD‐L1 expression in breast cancer could provide a new strategy for breast cancer treatment. Here, we demonstrate that moesin (MSN) phosphorylation by Rho‐associated protein kinase (ROCK) stabilizes PD‐L1 protein levels. Our results indicate that phosphorylated MSN may compete with the E3 ubiquitin ligase SPOP for binding PD‐L1. ROCK inhibition via the Y‐27632 inhibitor or MSN silencing decreased PD‐L1 expression, resulting in T‐cell activation both *in vitro* and *in vivo*. Administration of Y‐27632 into immunocompetent Balb/c mice bearing breast tumors suppressed tumor progression and enhanced CD4+ and CD8+ T‐cell infiltration. RNA‐seq analysis of Y‐27632‐treated mouse tumors revealed that ROCK inhibition upregulated several immune response genes. However, the combination of Y‐27632 and an anti‐PD‐1 antibody did not show additive or synergistic effects due to reduced PD‐L1 in the presence of Y‐27632. Our study unravels a previously unappreciated mechanism of PD‐L1 regulation through the ROCK‐MSN pathway. Moreover, we found that ROCK inhibitors could be combined with breast cancer immunotherapy.

## Graphical abstract

Here, we demonstrate that moesin (MSN) phosphorylation by Rho‐associated protein kinase (ROCK) stabilizes PD‐L1 protein levels by blocking SPOP mediating PD‐L1 degradation. Administration of ROCK inhibitor Y‐27632 into immunocompetent mice bearing breast tumors suppressed tumor progression and triggers antitumor T‐cell activity.

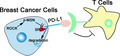

**Abbreviations**
CMTM4/6: CKLF‐like MARVEL transmembrane domain‐containing 4/6
ERM: ezrin, radixin, and moesin
GFP: green fluorescent protein
GO: Gene Ontology
IHC: immunohistochemistry
MSN: moesin
PARP: poly‐ADP‐ribose polymerase
PD‐1: programmed cell death protein 1
PD‐L1: programmed cell death ligand 1
PHA: phytohemagglutinin
PMA: phorbol myristate acetate
ROCK: rho‐associated protein kinase
SPOP: speckle‐type BTB/POZ protein

## 1 Introduction

Immune checkpoint inhibitors, such as programmed cell death ligand 1 (PD‐L1)/programmed cell death protein 1 (PD‐1) antibodies, have achieved significant clinical outcome in several cancer types [1, 2]. The application of anti‐PD‐L1 therapy is effective in the treatment of triple‐negative breast cancer [3]. The main challenges of applying anti‐PD(L)‐1 therapies in cancer treatment are low response rates and immune resistance [4]. Combination therapy of PD‐L1 and antitumor drugs is a reasonable strategy to improve anti‐PD‐L1 therapy efficiency. For example, a CDK4/6 inhibitor and anti‐PD‐1 immunotherapy enhanced tumor regression and overall survival rates of tumor‐borne mice [5], and blockade of PD‐L1 potentiated poly‐ADP‐ribose polymerase (PARP) inhibitor anticancer therapies [6]. Understanding the regulation of PD‐L1 expression will provide insights into new targets for combination therapy in breast cancer.

The PD‐L1 binds to PD‐1 in T cells, resulting in T‐cell anergy and apoptosis [7], while the efficiency of immunotherapy is closely associated with the number and activity of intratumoral CD8^+^ T cells [8]. PD‐L1 is induced by several cytokines, such as type I and type II interferons, tumor necrosis factor α, and vascular endothelial growth factor. The 3′‐UTR of PD‐L1 mRNA is usually disrupted in multiple cancers, leading to downregulation of the PD‐L1 transcriptional level [9]. As a transmembrane protein, PD‐L1 expression is extensively regulated by post‐translational modifications, such as phosphorylation, glycosylation, acetylation, palmitoylation, and ubiquitination [10, 11]. Additionally, membrane‐associated proteins CMTM6 and CMTM4 were recently identified to stabilize the membrane PD‐L1 level by preventing PD‐L1 from lysosome‐mediated degradation [12, 13].

Moesin (MSN) belongs to the ezrin, radixin, and moesin (ERM) family. ERM plays a pivotal role in regulating cell surface structures, such as microvilli and membrane ruffles, and specialized membrane domains [14]. With regard to biochemical characteristics, MSN consists of an FERM (4.1 protein, ezrin, radixin, and moesin) domain in the amino terminus and an ERM‐association domain (C‐ERMAD) in the C terminus, which is capable of binding to the FERM domain or F‐actin. In inactive conformation, the N‐terminal FERM domain binds to the C‐terminal region, while in active conformation, phosphorylation mediates the release of the FERM domain to interact with membrane proteins, such as CD44 and CD43 [15, 16]. In the disease setting, interaction of MSN and CD44 promotes glioma progression [16]. Activation of MSN is regulated by several kinases, including rho‐associated protein kinase (ROCK), which phosphorylates MSN on threonine 558 (Thr558) to inhibit intermolecular head‐to‐tail association [17]. Clinical trials have found that inhibition of the ROCK pathway contributes to cardiovascular benefits of statin therapy. Furthermore, ROCK inhibitors may also inhibit tumor metastasis [18]. More recently, they have been studied for the treatment of glaucoma [19].

In view of the limited understanding of regulation of PD‐L1 in breast cancer, we utilized mass spectrometry to screen for PD‐L1 interaction protein and found that MSN interacts with and regulates PD‐L1 expression. Moreover, we elucidated that ROCK‐mediated MSN Thr558 phosphorylation was required for PD‐L1 stabilization. In vivo studies proved that ROCK inhibitors achieved an antitumor activity similar to that of the anti‐PD‐1 antibody by stimulating T‐cell activity.

## 2 Materials and methods

### 2.1 Antibodies, cells, and chemicals

The following antibodies were used for western blot analyses, immunofluorescence, in vivo administration, and immunoprecipitation: PD‐L1 and MSN (Cell Signaling Technology, Danvers, MA, USA), phospho‐MSN (Abcam, Cambridge, UK), ROCK (Cell Signaling Technology, Danvers, MA, USA), FLAG (Sigma, St. Louis, MO, USA), and GFP (Invitrogen, Carlsbad, CA, USA). PD‐1 antibody (BE0146) for in vivo experiments was purchased from Bio X Cell (Lebanon, NH, USA). Y‐27632, cycloheximide, phorbol myristate acetate (PMA), and phytohemagglutinin (PHA) were purchased from MedChemExpress. The breast cancer cell lines MBA‐MD‐231 were ordered from the Cell Bank of Chinese Academy of Sciences, and BT549 was ordered from ATCC. The mouse breast cancer cell line EMT6 was ordered from ATCC. The authentication information is provided in supporting information (Appendix S1).

### 2.2 Plasmid construction and siRNA cell transfection

Human MSN, SPOP, and PD‐L1 cDNA were cloned in pCDNA3.1 with FLAG tag. PD‐L1 cDNA was subcloned into pEGFP‐N1 to generate GFP‐PD‐L1 construct. Small interfering RNAs (siRNAs) against the MSN, PD‐L1, and ROCK genes were transfected into cells using Lipofectamine 3000 (Life Technologies, Elmhurst, Illinois, USA) following the manufacturer’s instructions. The siRNA sequences were as follows:

MSN siRNA‐1: 5′‐GGAGGAUGCUGUCCUGGAAUA‐3′
MSN siRNA‐2: 5′‐GCUAAAUUGAAACCUGGAAUU‐3′
ROCK siRNA‐1: 5′‐GCACCAGUUGUACCCGAUUUA‐3′
ROCK siRNA‐2: 5′‐CGAUCGUCUCUAGGAUGAUAU‐3′
PD‐L1 siRNA: 5′‐CGAAUUACUGUGAAAGUCAAU‐3′

### 2.3 T‐cell killing assay

MDA‐MB‐231 cells were transduced with MSN siRNA or treated with Y‐27632 (20 μm). Jurkat T cells were activated by 25 ng·mL^−1^ of PMA and 1 μg·mL^−1^ of PHA for 24 h and then added to MDA‐MB‐231 cells at a Jurkat:MDA‐MB‐231 cell ratio of 5 : 1. The number of survived cells was captured under microscope and calculated.

### 2.4 PD‐L1 degradation assay

MBA‐MD‐231 cells were transfected with MSN siRNA and control siRNA. Forty‐eight hours later, the cells were treated with 50 mg·mL^−1^ of cycloheximide (CHX, Sigma‐Aldrich) for 0, 4, 8, 12, and 24 h. Cells were collected, the protein levels were determined by western blotting, and the subsequent quantification was performed with imagej software (NIH, USA).

### 2.5 Site‐directed mutagenesis

MSN T558A and T558D mutants were generated by site‐directed mutagenesis. Briefly, the primers used for mutagenesis were designed using the online QuikChange Primer Design Program provided by Agilent Technologies. The mutagenesis was performed using Pfu DNA polymerase (Agilent) and 300 ng plasmid template according to the manufacturer’s instruction. The PCR product was digested by DpnI endonuclease for 1 h at 37 °C, followed by transformation and sequencing.

### 2.6 Immunofluorescence staining

MDA‐MB‐231 cells were washed with PBS and fixed with 4% formaldehyde for 20 min, followed by permeabilization with Triton X‐100 for 5 min. Samples were blocked with 5% BSA for 20 min at R.T. and incubated with primary antibodies (p‐MSN, 1 : 200 dilution in 1% BSA; FLAG, 1 : 500 dilution in 1% BSA) overnight at 4 °C. After washing with PBS three times, secondary antibodies (donkey anti‐rabbit IgG Alexa Fluor 594 and donkey anti‐mouse IgG FITC from Invitrogen, 1 : 500 dilution in 1% BSA) were used for incubation for 1 h at room temperature. Then, cells were costained with DAPI (Invitrogen) and observed under a fluorescent microscope.

### 2.7 Immunohistochemistry (IHC)

Breast cancer tissue array (BC‐1601 and BC‐1602) was ordered from Iwill Biological Technology (Wuhan China). IHC was performed following the standard protocol as described previously. The first antibodies used were anti‐p‐MSN (1 : 100) and anti‐PD‐L1 (1 : 100). With regard to the histologic scores, the intensity of staining was classified into four groups: high (+++), medium (++), low (+), and negative (–).

### 2.8 Immunoprecipitation

MDA‐MB‐231 or BT‐549 cells were transfected with indicated plasmids using Lipofectamine^®^ 3000 (Invitrogen, Carlsbad, CA, USA), following the manufacturer’s instructions. After transfection for 48 h, cells were lysed in buffer (50 mm Tris/HCl, pH 7.4, 200 mm NaCl, 0.2% NP40, 10% glycerol, 1 mm NaF, and complete protease inhibitor cocktail, Roche). The cell extracts were incubated with antibody at 4 °C overnight. Then, protein A/G magnetic beads were added for 2 h at 4°C. After washing with lysis buffer thrice, the immunoprecipitates were subsequently boiled with 2× Laemmli buffer and analyzed using western blotting.

### 2.9 Breast tumor mouse model

The animal studies were performed under the approval of Tianjin Medical University Cancer Institute and Hospital Animal Care and Use Committee (Approved No. LLSP2019004). Breast tumor mouse model and treatment protocol were performed as previously described with a few modifications [6]. Briefly, EMT6 (1 × 10^5^) cells in Matrigel were injected into mammary fat pad of BALB/c mice (*n* = 8 per group). Mice were injected intraperitoneally with 8 mg·kg^−1^ Y‐27632 or saline daily from day 5 after implantation, and 75 mg anti‐mouse PD‐1 antibody (BE0146, Bio X Cell) or control rat IgG2b every 4 days from day 8 after implantation. All mice were terminated at day 28. Tumor volumes were measured with a digital caliper every 3 days and calculated using the formula: 1/2 × length × width^2^.

### 2.10 IL‐2 production assay

MDA‐MB‐231 cells were transduced with MSN siRNA or treated with Y‐27632 (20 μm). Jurkat T cells were activated by 25 ng·mL^−1^ of PMA and 1 μg·mL^−1^ of PHA for 24 h and then added to MDA‐MB‐231 cells at a Jurkat:MDA‐MB‐231 cell ratio of 4 : 1. The cell culture media were harvested 72 h after coculture. The IL‐2 level in culture media was measured using IL‐2 ELISA kit according to the manufacturer’s instructions.

### 2.11 RNA‐seq analysis

Total tumor RNA was isolated and used for RNA‐seq analysis. cDNA library was constructed and sequenced using the Illumina HiSeq platform (Genewiz, Suzhou, China). High‐quality reads were aligned to the human reference genome (GRCh38) using Bowtie 2. Differential genes showed in the Venn diagram were generated using the tool Venny 2.1. The expression levels for each of the genes were normalized to fragments per kilobase of exon model per million mapped reads.

### 2.12 Statistical analysis

The Student *t*‐test or one‐way analysis of variance was used to compare experimental data. Statistical analysis and plot were performed using the graphpad (San Diego, CA, USA) software. image‐pro plus (rockville, maryland, usa) software was used to semiquantify IHC data, and the Pearson chi‐squared test was used for statistical analysis. A *P*‐value < 0.05 was considered statistically significant.

## 3 Results

### 3.1 PD‐L1 expression is regulated by MSN in breast cancer

**Fig. 1:**
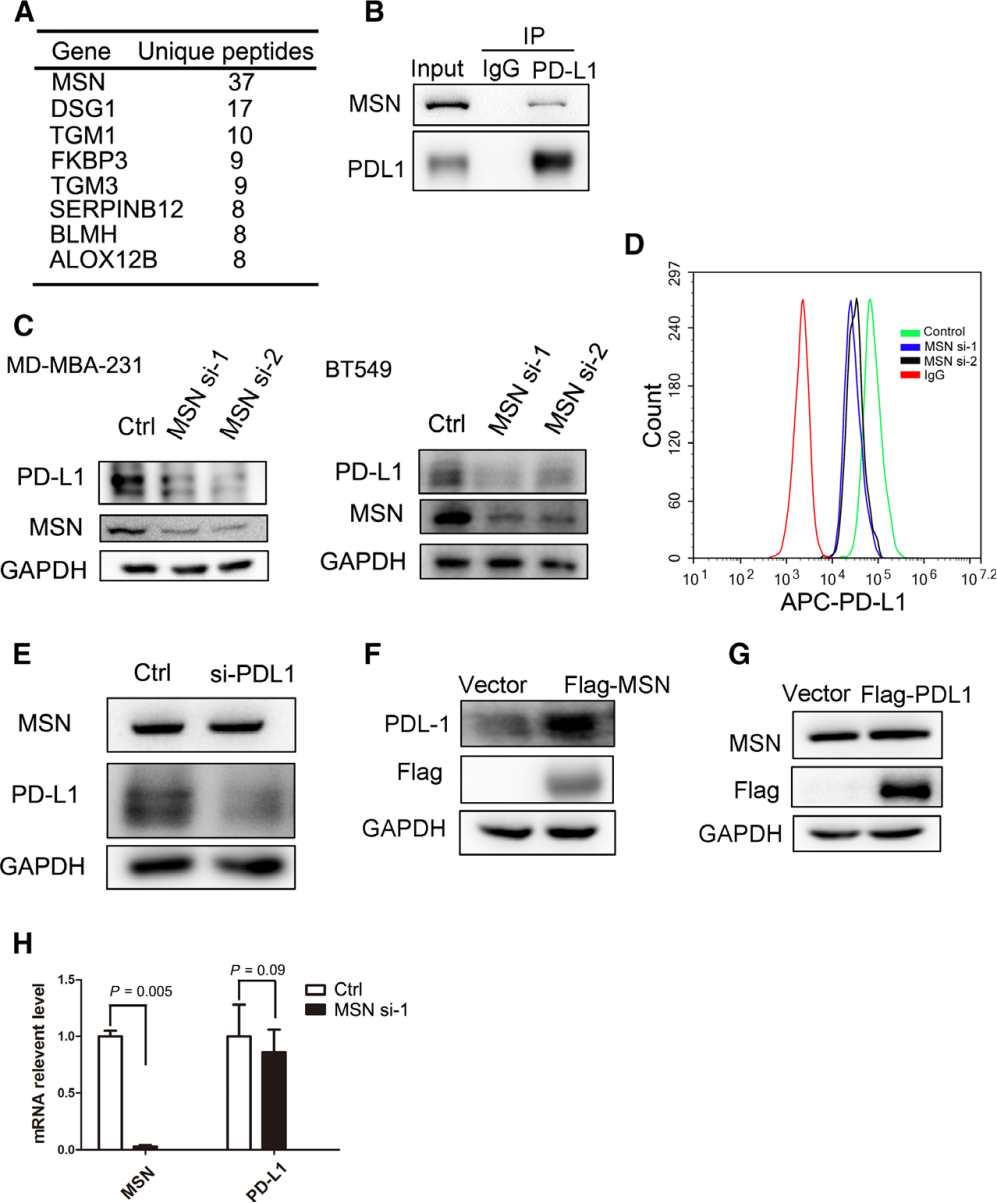
Regulation of PD‐L1 by MSN in breast cancer cell. (A) Mass spectrometry of the FLAG‐PD‐L1 immunoprecipitate, and enriched interacting proteins were listed. (B) Immunoblot of PD‐L1 co‐immunoprecipitation using indicated antibodies (*n* = 3). (C) PD‐L1 level expression after silencing MSN with siRNAs in MDA‐MB‐231 cells or BT‐549 (*n* = 3). (D) The cell surface PD‐L1 level of MSN‐silenced cells was analyzed by FACS with an APC‐conjugated antibody (*n* = 3). (E), Immunoblot analysis of WCL from MDA‐MB‐231 cells transfected with control or PD‐L1 siRNAs (*n* = 3). (F), Immunoblot analysis of WCL from MDA‐MB‐231 cells transfected with vector or FLAG‐MSN (*n* = 3). (G), Immunoblot analysis of WCL from MDA‐MB‐231 cells transfected with FLAG‐MSN (*n* = 3). (H), qPCR analysis of the PD‐L1 mRNA level from MDA‐MB‐231 cells transfected with control or MSN siRNAs. Standard error of mean (SEM) from triplicates is shown by vertical bars (*n* = 3). Statistical significance was assessed by Student’s *t*‐test.

To identify regulators of PD‐L1, we immunoprecipitated FLAG‐tagged PD‐L1 from MDA‐MB‐231 breast cancer cells and screened for interacting proteins by mass spectrometry (Fig. 1A). An additional Excel file shows this in more detail (see Appendix S2). The top enriched protein MSN interaction with PD‐L1 is confirmed by co‐immunoprecipitation (Fig. 1B). Further, we found that knocking down MSN in two breast cancer cell lines (MDA‐MB‐231 and BT‐549) reduced the PD‐L1 level (Fig. 1C). Using fluorescence‐activated cell sorting (FACS), we demonstrated that cell surface PD‐L1 level decreased after silencing MSN (Fig. 1D). However, the MSN protein level did not change after silencing PD‐L1 expression (Fig. 1E). Additionally, MSN overexpression increased the PD‐L1 level (Fig. 1F), but MSN level did not change after PD‐L1 overexpression (Fig. 1G), suggesting that MSN is an upstream protein for PD‐L1. The PD‐L1 mRNA level did not change after silencing MSN (Fig. 1H). Generally, our data demonstrate that MSN positively regulates PD‐L1 protein expression via a translational or post‐translational mechanism.

### 3.2 MSN competes with SPOP to prevent PD‐L1 degradation

**Fig. 2:**
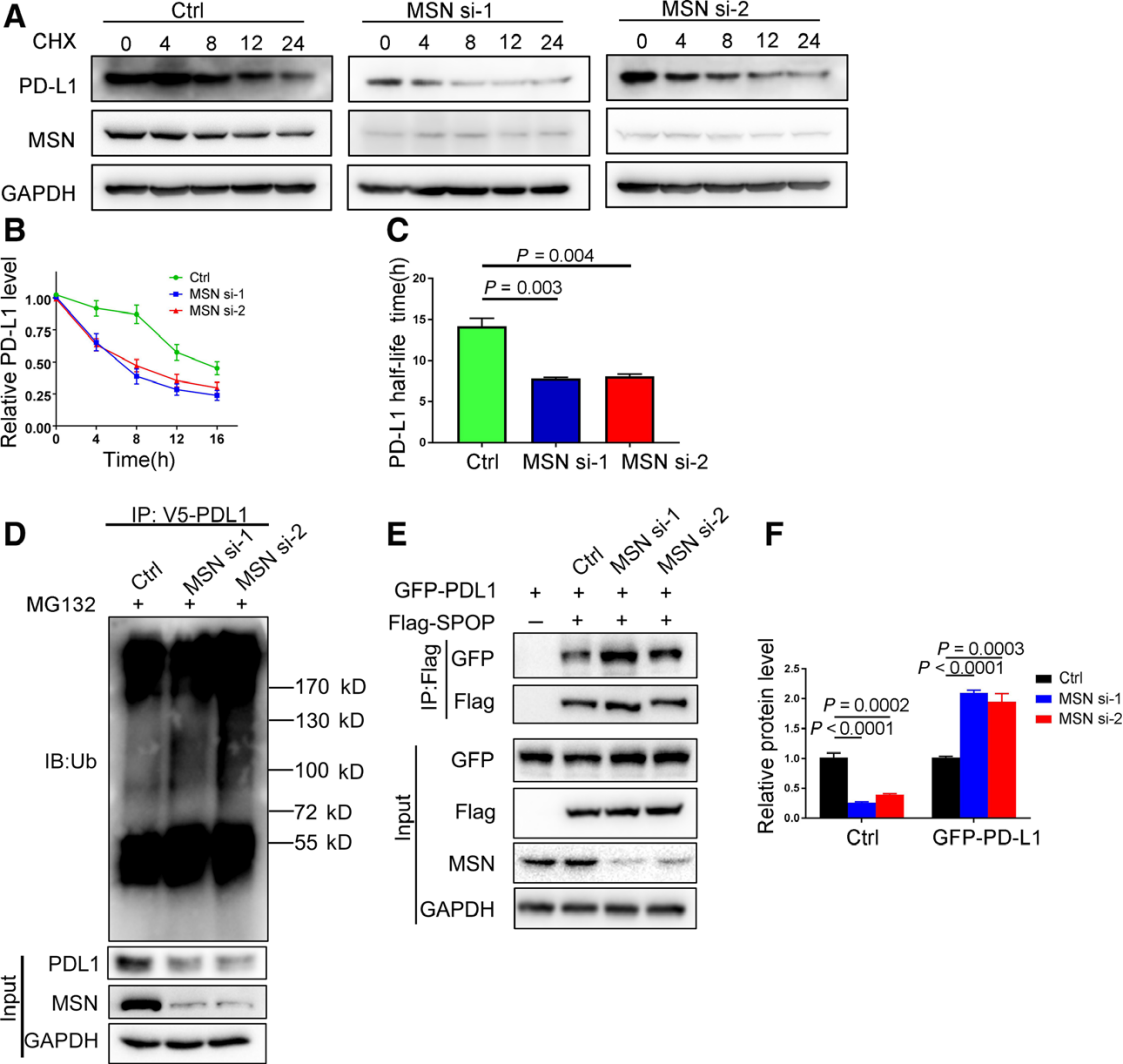
MSN regulates ubiquitination of PD‐L1. (A) MDA‐MB‐231 cells were transfected with MSN siRNAs and treated with cycloheximide (Chx, 50 μg·mL^−1^) for indicated time points (*n* = 3). (B) The PD‐L1 level was shown by western blotting and semiquantified by imagej software. Standard error of mean (SEM) from triplicates is shown by vertical bars (*n* = 3). Statistical significance was assessed by Student’s *t*‐test. (C) The mean degradation rate t1/2 was calculated and analyzed statistically. Standard error of mean (SEM) from triplicates is shown by vertical bars (*n* = 3). Statistical significance was assessed by Student’s *t*‐test. (D) Immunoblot of MDA‐MB‐231 cells transfected with control or MSN siRNAs and treated with MG132 (10 μm) for 6 h (*n* = 3). (E) MDA‐MBE‐231 cells were transfected with control or MSN siRNAs, GFP‐PD‐L1, and FLAG‐SPOP as indicated. Immunoprecipitation of SPOP was performed using an anti‐FLAG antibody and blot with a GFP antibody (*n* = 3). (F) The relative protein levels of MSN and GFP‐PD‐L1 from PD‐L1‐SPOP co‐IP repeats were calculated by imagej software and analyzed statistically. Standard error of mean (SEM) from triplicates is shown by vertical bars (*n* = 3). Statistical significance was assessed by Student’s *t*‐test.

We investigated how MSN mediated PD‐L1 expression. Using the cycloheximide chasing assay, we found that silencing MSN enhanced the degradation rate of PD‐L1 (Fig. 2A). We have calculated mean t1/2 values from several repeats and analyzed the data statistically (Fig. 2B,C). Furthermore, the amount of ubiquitinated PD‐L1 increased in the absence of MSN, suggesting that MSN may protect PD‐L1 from ubiquitination or disrupt proteasomal degradation of PD‐L1 (Fig. 2D). SPOP is a major E3 ligase adaptor for PD‐L1 degradation [5]. We found that the interaction of PD‐L1 with SPOP was enhanced after MSN depletion (Fig. 2E,F), indicating that MSN competes with SPOP to bind to PD‐L1. Therefore, our data support the notion that MSN interacts with PD‐L1 and contributes to the stability of PD‐L1 by blocking the interaction of PD‐L1 and SPOP.

### 3.3 ROCK‐dependent MSN Thr558 phosphorylation upregulates PD‐L1

**Fig. 3:**
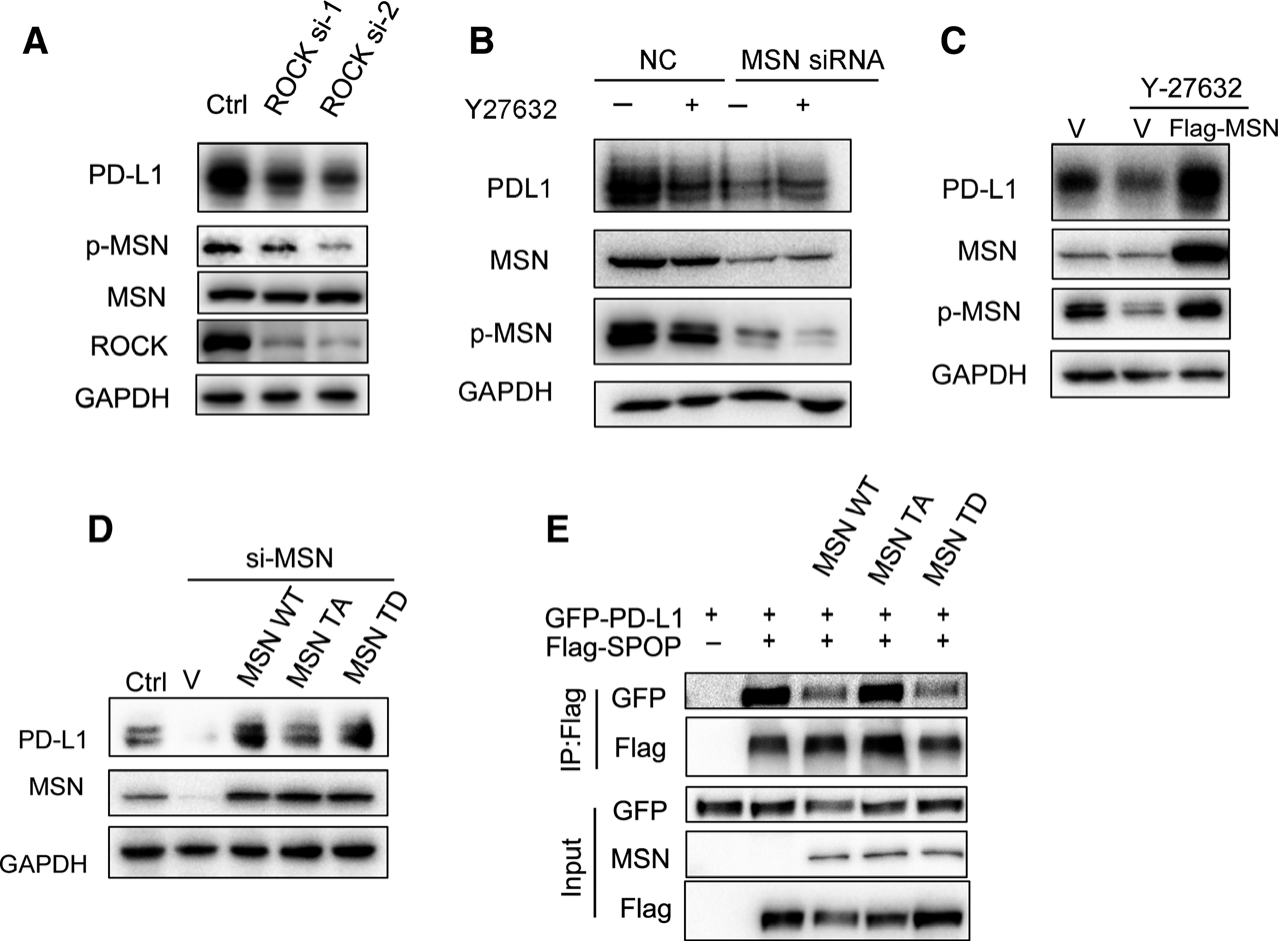
Phosphorylation of MSN is required for stabilizing PD‐L1. (A) Immunoblot analysis of WCL from MDA‐MB‐231 cells transfected with control of ROCK siRNAs (*n* = 3). (B) Immunoblot analysis of WCL from MDA‐MB‐231 cells transfected with control or MSN siRNAs and treated with Y‐27632 (20 μm) for 24 h (*n* = 3). (C) Immunoblot analysis of WCL from MDA‐MB‐231 cells transfected with vector or FLAG‐MSN and treated with Y‐27632 (20 μm) for 24 h (*n* = 3). (D) Western blot analysis showed PD‐L1 level was rescued in MSN‐silenced cells after re‐introducing vector, MSN‐T558D (TD), WT‐MSN, or MSN‐T558A (TA) (*n* = 3). (E) MDA‐MB‐231 cells were transfected with MSN‐WT, MSN‐T558D (TD), MSN‐T558A (TA), GFP‐PD‐L1, and FLAG‐SPOP as indicated. Immunoprecipitation of SPOP was performed using the anti‐FLAG antibody and blot with the GFP antibody (*n* = 3).

The functional activity of MSN is regulated by phosphorylation, and Thr558 is phosphorylated by the ROCK [17]. We sought to examine whether ROCK was required for PD‐L1 regulation. As shown, the phosphorylated MSN level decreased by siRNA silencing ROCK (Fig. 3A) or in the presence of the ROCK inhibitor Y‐27632 (Fig. 3B). As expected, the PD‐L1 level decreased after silencing ROCK or in the presence of Y‐27632 (Fig. 3A,B). To test whether PD‐L1 is regulated by ROCK via MSN, we knocked down MSN in the presence of Y‐27632 and found that the effect of PD‐L1 downregulation by Y‐27632 diminished (Fig. 3B). Moreover, when we overexpressed MSN in Y‐27632‐treated cells, the PD‐L1 level was restored (Fig. 3C). These results indicate that the PD‐L1 expression regulated by ROCK is dependent on MSN.

ROCK is a key kinase for MSN Thr558 phosphorylation during cell movement. Next, we expressed shRNA‐resistant, wild‐type (WT‐MSN), unphosphorylated (T558A‐MSN), or phosphor‐mimic (T558D‐MSN) MSN in MSN knockdown cells. We found that, while both WT‐MSN and T558D‐MSN fully restored PD‐L1 levels, T558A‐MSN expression can only partially restore it (Fig. 3D). Meanwhile, we found that there was colocalization of phospho‐MSN and PD‐L1 on the cell membrane by immunofluorescence microscopy (Fig. S1). Additionally, using Co‐IP analysis, we found that WT‐MSN and T558D‐MSN competed with SPOP to bind to PD‐L1, while T558A‐MSN did not (Fig. 3E).

### 3.4 Correlations of phosphorylated MSN (p‐MSN) and PD‐L1 expression in breast cancer tissues

**Fig. 4:**
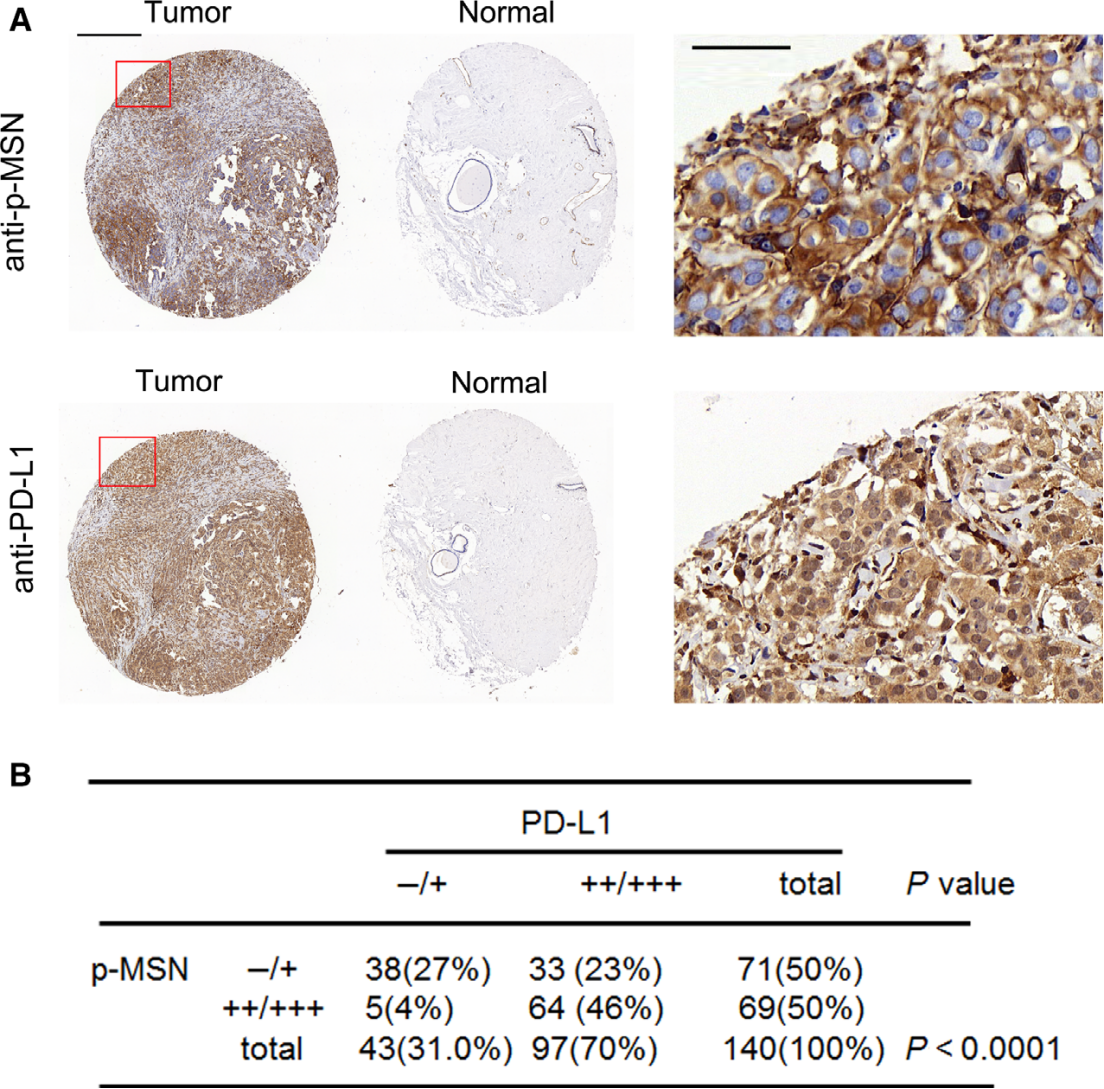
Correlation between p‐MSN and PD‐L1 in clinical breast cancer tissue array. (A) Representative images of IHC staining of p‐MSN and PD‐L1 in human breast cancer tissues (*n* = 140) and normal tissues. Scale bar: 1000 μm (left), 50 μm (right). (B) Correlation between p‐MSN and PD‐L1 was analyzed using the Pearson chi‐squared test (*P* < 0.0001).

To explore the clinical relevance of p‐MSN and PD‐L1, we checked expression of p‐MSN and PD‐L1 in a breast cancer tissue array by IHC (Fig. 4A). In addition, we used negative control for testing antibody specificity (Fig. S2). High p‐MSN level was detected in 69 (50.2%) of 140 specimens, of which 97 cases (70.0%) showed high PD‐L1 expression (Fig. 4B). The Pearson chi‐squared (χ^2^) test further showed that a positive correlation between p‐MSN level and PD‐L1 expression existed in human breast cancer specimens. The clinical information with regard to breast cancer subtypes, stages were summarized and attached (Appendices S3 and S4).

### 3.5 Inhibition of the ROCK‐MSN pathway represses tumor progression through downregulation of PD‐L1

**Fig. 5:**
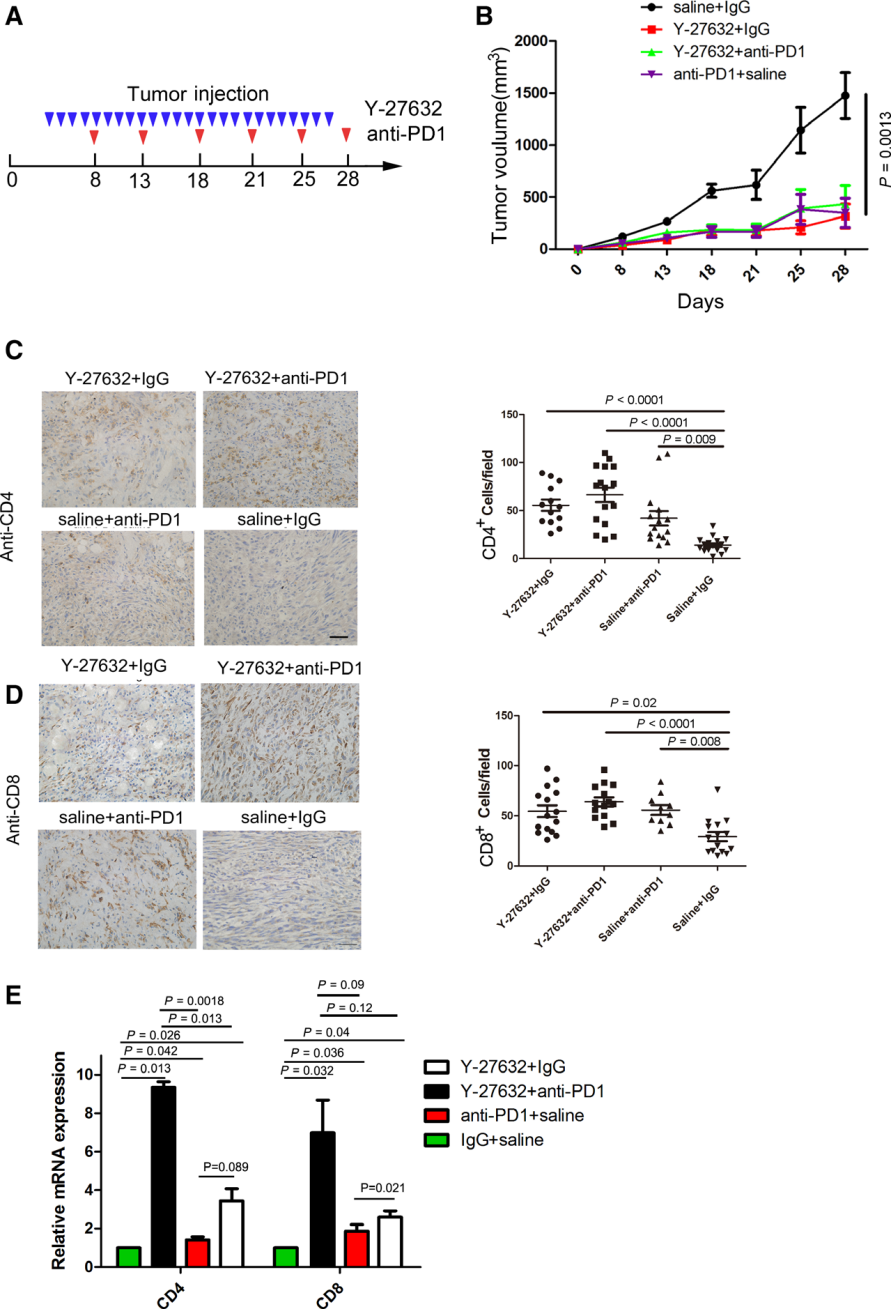
The ROCK inhibitor Y‐27632 represses breast cancer progression. (A) Effects of Y‐27632 (8 mg·kg^−1^) and/or anti‐PD‐1 antibody (75 μg) treatment on tumor growth in the EMT6 syngeneic mouse model (*n* = 8). (B) The tumor was collected after Y‐27632 and/or anti‐PD‐1 antibody treatment at the indicated time points in the EMT6 syngeneic mouse model. IHC staining of CD4 (C) and CD8 (D) of tumors harvested after Y‐27632 and/or anti‐PD‐1 antibody treatment. Scale bar: 100 μm. (E) qPCR analysis of CD4 and CD8 from tumors after Y‐27632 and/or anti‐PD‐1 antibody treatment. Standard error of mean (SEM) from triplicates is shown by vertical bars (*n* = 3). Statistical significance was assessed by Student’s *t*‐test.

In light of the evidence that ROCK‐MSN mediates PD‐L1 expression, we hypothesized that inhibition of the ROCK‐MSN pathway might stimulate the tumor immune response. To test this, we established a breast cancer tumor model in immunocompetent mice using the murine breast cancer cell line EMT6 [6]. The ROCK inhibitor Y‐27632 or PD‐1 antibody was injected into tumor‐borne mice separately or in combination (Fig. 5A). Administration of Y‐27632 or anti‐PD‐1 antibody significantly repressed tumor growth. However, the combination of Y‐27632 and anti‐PD‐1 antibody demonstrated no additive/synergism activity compared to each treatment alone (Fig. 5A,B), suggesting that Y‐27632 and anti‐PD‐1 treatment may share a similar mechanism. The IHC assay revealed that administration of Y‐27632 reduced the PD‐L1 protein level (Fig. S3). Analysis of tumor‐infiltrating lymphocytes (TILs) by IHC staining showed that Y‐27632‐treated or anti‐PD‐1 antibody‐treated tumor had significantly increased CD4^+^ and CD8^+^ T‐cell infiltration (Fig. 5C,D). We also found that CD4 and CD8 mRNA levels significantly increased after Y‐27632 and/or anti‐PD‐1 antibody treatment (Fig. 5E). There was no significant difference in CD4 and CD8 mRNA levels between Y‐27632 and anti‐PD‐1 antibody treatment. CD4 mRNA levels significantly increased after Y‐27632 plus anti‐PD‐1 antibody treatment compared to Y‐27632 or anti‐PD‐1 antibody treatment. However, there were no significant differences in CD8 mRNA levels among Y‐27632 and/or anti‐PD‐1 antibody treatments. These data suggest that inhibition of the ROCK‐MSN pathway represses tumor progression by enhancing TILs.

### 3.6 ROCK inhibitor Y‐27632 activates immune response genes and enhances T‐cell activity

**Fig. 6:**
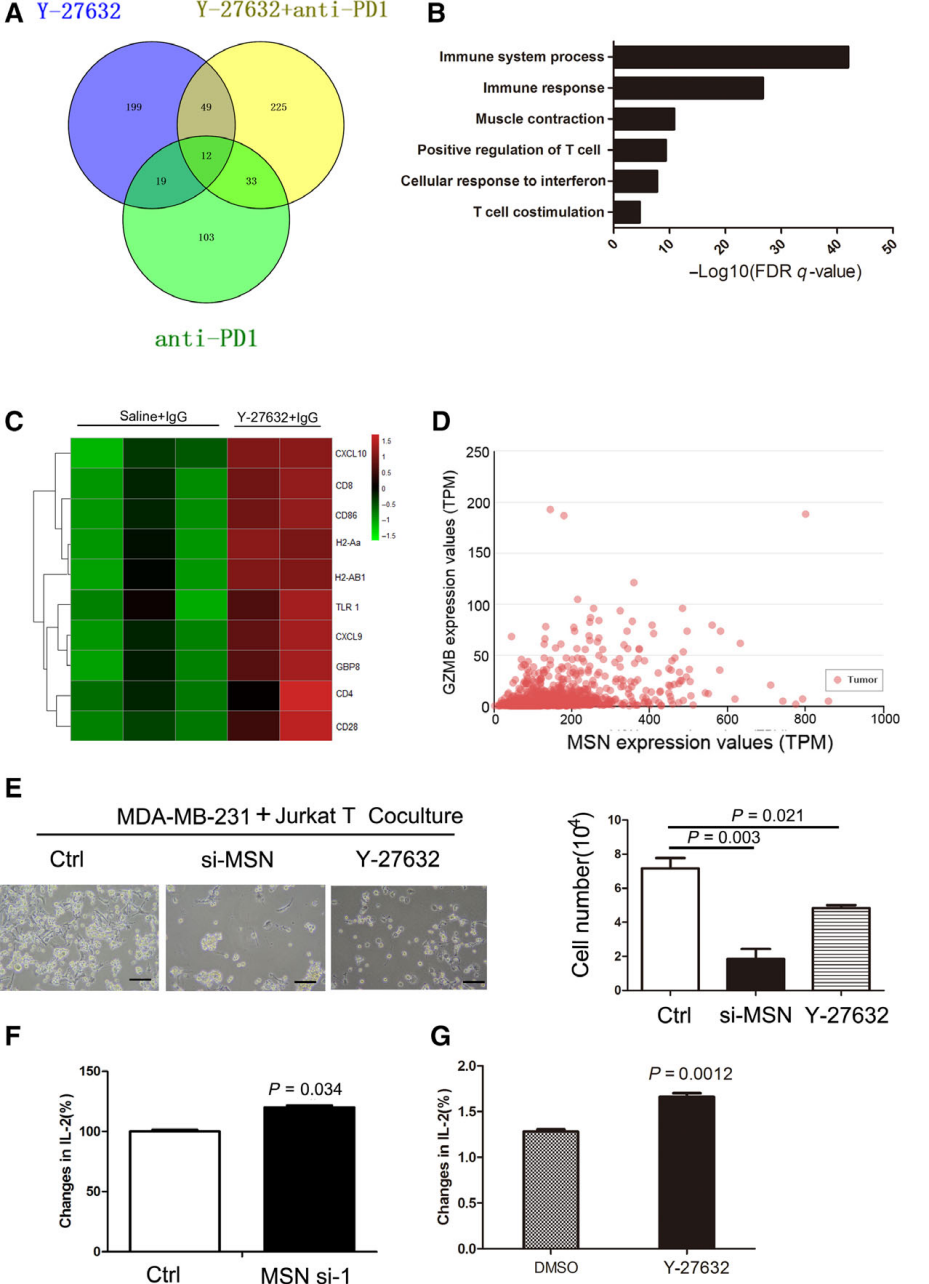
Administration of ROCK inhibitor elicits the immune response. Murine tumors after Y‐27632 and/or anti‐PD‐1 antibody treatment were harvested for transcriptomic analysis. (A) The Venn diagram shows differential genes from Y‐27632‐treated and/or anti‐PD‐1 antibody‐treated tumors. Three tumor samples from each group were used (*n* = 3). (B) The GO pathway analysis of Y‐27632‐treated tumors or control. Three tumor samples from each group were used (*n* = 3). (C) Heat map showing expression value (z‐score based on cufflink count) of Y‐27632‐treated tumors (*n* = 2) or control (*n* = 3). (D) The correlation expression of GZMB with MSN in TCGA dataset was analyzed using the online tool Ulcan. Breast invasive carcinoma samples were analyzed (*n* = 1097). (E) Survived cell number of MDA‐MB‐231 cells after 3‐day cocultures of Jurkat T cells. Standard error of mean (SEM) from triplicates is shown by vertical bars (*n* = 3). Statistical significance was assessed by Student’s *t*‐test. Scale bar: 100 μm. (F) IL‐2 levels in supernatants from 3‐day cocultures of Jurkat T cells and MDA‐MB‐231 cells transfected were detected by the ELISA. *P < 0.05, unpaired two‐tailed *t*‐test. Standard error of mean (SEM) from triplicates is shown by vertical bars (*n* = 3). (G) IL‐2 levels in supernatants from 3‐day cocultures of Jurkat T cells and MDA‐MB‐231 cells treated with DMSO or Y‐27632 (20 μm) were detected by the ELISA. Standard error of mean (SEM) from triplicates is shown by vertical bars (*n* = 3). Statistical significance was assessed by Student’s *t*‐test.

To further explore the mechanism of ROCK inhibition in tumor suppression, we performed RNA‐seq of each treated tumor and analyzed the transcriptional regulation network. We used the EMT6 mouse breast cancer line grown on the BALB/c mouse model. The Venn diagram showed differential expressing genes in Y‐27632‐treated and/or anti‐PD‐1 antibody‐treated tumors (Fig. 6A). By analyzing differential genes after Y‐27632 treatment by GO analysis, we found that Y‐27632 significantly induced the immune response and positively regulated T‐cell pathways (Fig. 6B). The cluster alignment analysis revealed that Y‐27632‐treated tumors highly expressed immune response genes (Fig. 6C). We have performed qPCR validation of several identified genes including CXCL10, H2Aa, and CD86 in the cells. In consistent with the RNA‐seq data, the results showed CXCL10, H2Aa, and CD86 expression levels were significantly upregulated in response to Y‐27632 (Fig. S4). Representative genes are those positively regulating the proliferation of T cells. These data imply that downregulation of PD‐L1 by Y‐27632 improves tumor immune cell infiltration. To further study the relationship between MSN and immune response, we analyzed MSN and GZMB expression in breast cancer samples from The Cancer Genome Atlas database [20]. GZMB is an important biomarker for cytotoxic activity of T and NK cells. Gene correlation analysis revealed that MSN and GZMB were positively correlated with a Pearson correlation coefficient of 0.37 (Fig. 6D). To study the role of ROCK‐MSN pathway in T‐cell activation, we cocultured Jurkat T and MDA‐MB‐231 cells and found more T‐cell‐mediated cell death when silencing MSN expression or inhibition of the ROCK by Y‐27632 (Fig. 6E). Furthermore, silencing MSN by siRNA or inhibition of ROCK by Y‐27632 significantly increased the secretion of IL‐2 of Jurkat cells (Fig. 6F). It is noted that Y‐27632 had a limited effect on the proliferation and apoptosis of T cells (Fig. S5A,B). Generally, these data indicate that inhibition of the ROCK‐MSN pathway improves T‐cell activation via downregulation of PD‐L1.

## 4 Discussion

PD‐L1 expression is closely correlated with tumor immune microenvironment especially in CD8+ T‐cell infiltration [8]. Despite the promising results of PD‐L1‐based immunotherapy, a large number of patients do not respond to the treatment. Therefore, understanding PD‐L1 regulation is critical for combining immunotherapy and chemotherapy.

Previously, membrane‐associated proteins CMTM6 and CMTM4 were identified to stabilize the membrane PD‐L1 level by preventing PD‐L1 from lysosome‐mediated degradation [11]. Genome‐wide screening found that the 3′ region of the PD‐L1 gene was commonly disrupted by structural variations, leading to tumor growth and immune evasion [9]. Low PD‐L1 expression in tumor cells and high intratumoral CD8+ T‐cell content correlated positively with patient survival [8, 11, 20]. More importantly, the PD‐L1/PD‐1 pathway blockade suppresses tumor progression and metastasis [21]. Our findings reveal that MSN competes with SPOP to avoid E3 ubiquitin ligase‐mediated PD‐L1 degradation. How this mechanism interacts with CMTM‐4 and CMTM‐6 is still unknown. It is likely that CMTM‐4 and CMTM‐6 may share a similar molecular mechanism with MSN to modulate PD‐L1 expression. However, regulation of the PD‐L1 level mediated by MSN required phosphorylation modification, which makes phosphorylated MSN to be a more plausible therapeutic target than CMTM‐4 and CMTM‐6.

Clinical data show that the crosstalk between cancer and T cells is extremely important in the efficacy of anti‐PD‐1/PD‐L1‐based immunotherapy. T cells secrete interferon gamma and stimulate expression of PD‐L1 of neighboring cancer cells, which hinders the PD‐1 ligand on T cells, resulting in T‐cell anergy and apoptosis [22]. Reduction of PD‐L1/PD‐1 signaling by downregulation of PD‐L1 expression via CMTM‐4 and CMTM‐6 leads to the activation of T cells, with the feature of increasing secretion of IL‐2 and high killing activity [3, 21]. Our data show that silencing MSN or inhibiting MSN phosphorylation by Y‐27632 enhanced the secretion of IL‐2 of Jurkat T cells cocultured with MDA‐MB‐231 cells, indicating silencing MSN modulating T‐cell activity via the PD‐L1‐PD‐1 signaling pathway. Further, we observed that the administration of Y‐27632 in breast cancer mice increased CD4+ CD8 + T‐cell infiltration in tumor, indicating that T‐cell infiltration contributes to tumor regression.

As a cytoskeleton‐associated protein, MSN, together with other ERM protein members ezrin and radixin, regulates cell cortex, membrane blebs, and receptor complex [14, 23, 24]. A previous study showed that MSN was frequently overexpressed in high‐grade glioblastoma, and MSN interacted and colocalized with CD44 [16]. These reports indicate that MSN could be associated with membrane proteins, such as PD‐L1. Furthermore, our data demonstrate the phosphorylated MSN was required to stabilize PD‐L1.

A number of inhibitors have recently shown to enhance immunotherapy outcome by modulating PD‐L1 expression. For example, PARP1 inhibitors upregulated PD‐L1 expression via inactivating GSK‐3β. The combination treatment of PARP1 inhibitor and anti‐PD‐1 antibody increased the therapeutic effect in a mouse model [6]. A CDK4/6 inhibitor increased PD‐L1 protein levels by suppressing CDK4‐mediated phosphorylation of SPOP [5]. Our data demonstrate that treatment with the ROCK inhibitor Y‐27632 significantly inhibited breast cancer growth of EMT6 transplanted mice, and the effect was comparable with that of anti‐PD‐1 antibody treatment. In our case, inhibition of ROCK‐MSN pathway only by Y‐27632 showed similar activity compared to anti‐PD‐1 treatment, which resulted in T‐cell activation and tumor growth regression. However, it is not surprising that the combination of Y‐27632 and anti‐PD‐1 antibody treatment did not show more therapeutic benefit, due to their similar mechanism. In order to observe the combination effect, low dose of each inhibitor may be applicable to use. However, via an intraperitoneal route, lower dosages of inhibitor administration may result in insufficient serum concentrations to reach target tumor tissues. Regardless, single‐dose effects have limitations, and multiple dose combination needs to be tested in future study. A previous study showed that inhibition of ROCK by administration of Y‐27631 in a human breast cancer‐immunodeficient (NOD/SCID) mouse model reduced the tumor growth; however, the effect was not significant [25]. Our study provides an explanation for this occurrence as Y‐27631 has to activate the immune response to achieve an antitumor effect. Another group also reported a similar antitumor activity of ROCK inhibitor Y‐27632 in a syngeneic tumor model [26]. Regarding the mechanism, they demonstrated that ROCK blockade enhanced tumor cell phagocytosis and promoted T‐cell priming. In our case, we found increased CD4+ and CD8+ T‐cell infiltration in Y‐27632‐treated tumor, which is consistent with the result of a previous study. In our studies, we provide multiple lines of evidence to prove the mechanism of PD‐L1 regulation by the ROCK‐MSN pathway. More interestingly, PD‐1/PD‐L1 was shown to inhibit macrophage phagocytosis in the tumor microenvironment [27], indicating multiple roles of the ROCK inhibitor in suppressing tumor progression by modulating tumor immunity microenvironment.

The use of Y‐27632 has been tested to promote corneal endothelial wound healing in a phase I clinical study [28]. Our findings suggest that Y‐27632 may be used for further clinical study of immunotherapy.

## 5 Conclusions

Using proteomic methods, we identified MSN as the top enriched interacting protein of PD‐L1 in breast cancer. Furthermore, we provide evidence that PD‐L1 expression is regulated by ROCK phosphorylation of MSN Thr558. Administration of a ROCK inhibitor suppresses tumor progression and elicits the immune response in tumor microenvironment through downregulation of PD‐L1. These results highlight the potential of using ROCK inhibitors in breast cancer treatment.

## Conflict of interest

The authors declare no conflict of interest.

## Author contributions

BX designed the experiments of this study. FM and YS conducted the experiments, data analysis, and critical discussions of the results. All authors contributed to the writing and editing of the manuscript and approved the final draft of the manuscript.

## Supporting information


**Fig. S1.** (A) Immunofluorescence of Flag‐PD‐L1 and p‐MSN in MDA‐MB‐231 cells. A representative experiment (n=3) is shown. Scale bar: 10μm.
**Fig. S2.** (A) IHC staining of PD‐L1 from murine tumors after DMSO or Y‐27632 treatment. A representative experiment (n=3) is shown. Scale bar: 100μm.
**Fig. S3.** Representative image from IHC staining of tissue array without first antibody staining as negative control. A representative experiment (n=3) is shown. Scale bar: 100μm.
**Fig. S4.** qPCR analysis of CXCL10, H2Aa and CD86 in cells after Y‐27632 treatment or control. Standard error of mean (SEM) from triplicates are shown by vertical bars (n=3).
**Fig. S5.** (A) Cell proliferation assay after Y‐27631 treatment. Standard error of mean (SEM) from triplicates are shown by vertical bars (n=3). (B) Cells apoptosis after Y‐27631 treatment for 3 days were detected by FACS using Annexin V‐FITC/PI double staining. A representative experiment (n=3) is shown.

Appendix S1

Appendix S2

Appendix S3

Appendix S4

## Data Availability

All data generated or analyzed during this study are included in this published article and its supplementary information files. Gene correlation assay in TCGA dataset was performed by online tool Ulcan (http://ualcan.path.uab.edu/analysis.html).
