# Supplementary figures and images for: Rho‐associated protein kinase‐dependent moesin phosphorylation is required for PD‐L1 stabilization in breast cancer
Source: Mol Oncol. 2020 Oct 3;14(11):2701–12. doi: 10.1002/1878-0261.12804 (PMC7607174; doi:10.1002/1878-0261.12804)

A

Fig.S1

Flag-PD-L1

p-MSN

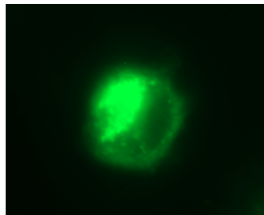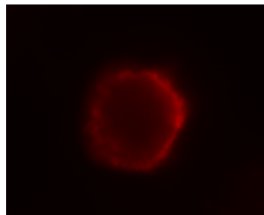

DAPI

Merge

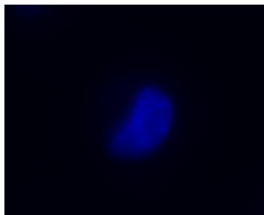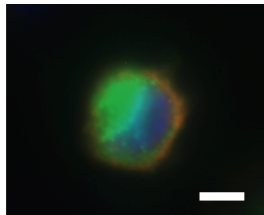

A

Fig.S2

Y27632

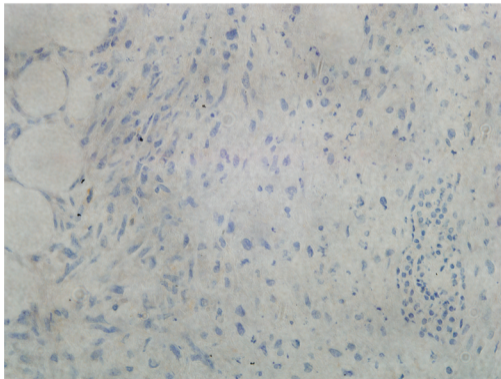

Saline

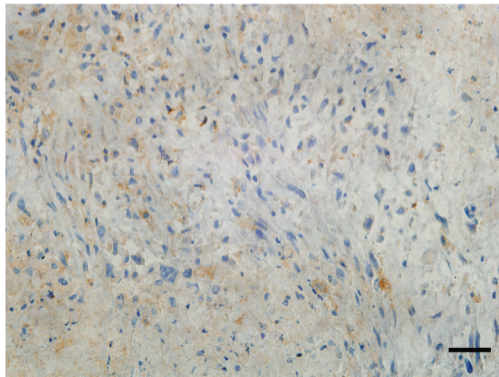

Fig.S3

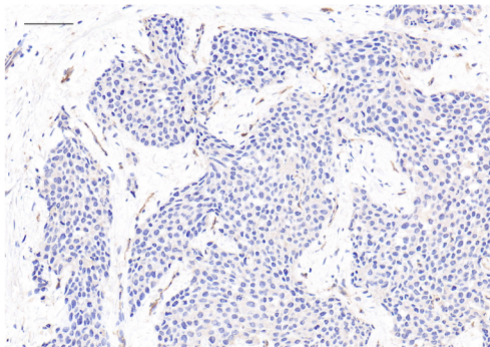

Fig.S4

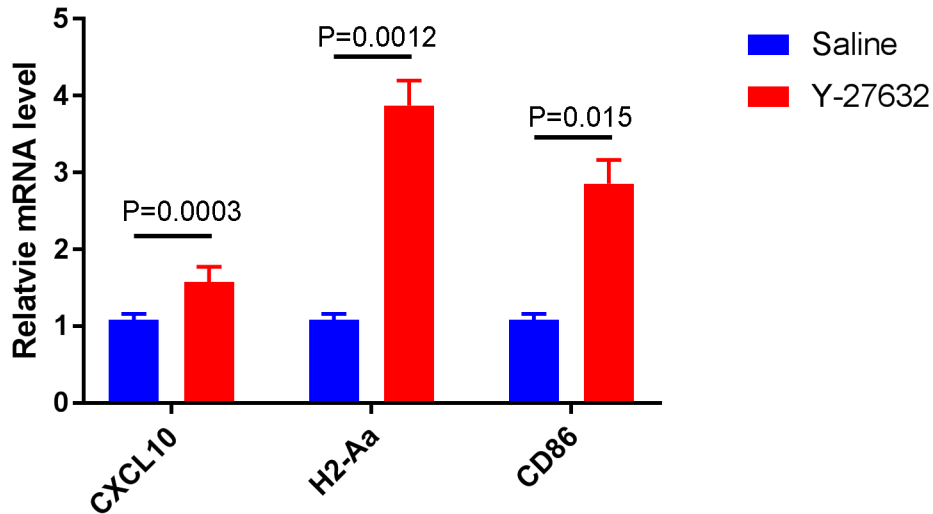

Fig.S5

A

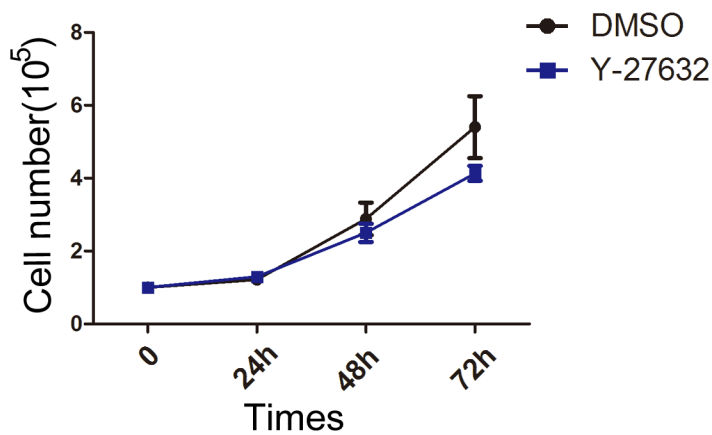

B

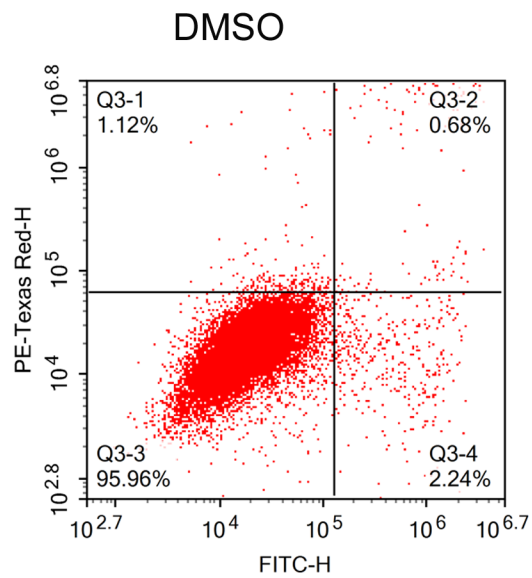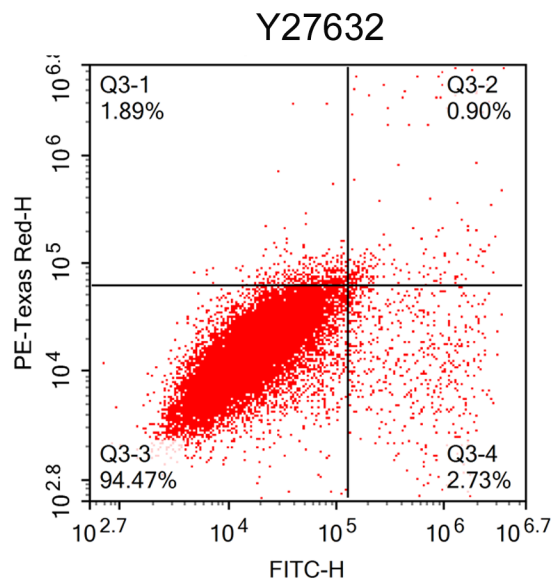

Supplement: Supplementary file 1 — Fig. S1. (A) Immunofluorescence of Flag‐PD‐L1 and p‐MSN in MDA‐MB‐231 cells. A representative experiment (n=3) is shown. Scale bar: 10μm. Fig. S2. (A) IHC staining of PD‐L1 from murine tumors after DMSO or Y‐27632 treatment. A representative experiment (n=3) is shown. Scale bar: 100μm. Fig. S3. Representative image from IHC staining of tissue array without first antibody staining as negative control. A representative experiment (n=3) is shown. Scale bar: 100μm. Fig. S4. qPCR analysis of CXCL10, H2Aa and CD86 in cells after Y‐27632 treatment or control. Standard error of mean (SEM) from triplicates are shown by vertical bars (n=3). Fig. S5. (A) Cell proliferation assay after Y‐27631 treatment. Standard error of mean (SEM) from triplicates are shown by vertical bars (n=3). (B) Cells apoptosis after Y‐27631 treatment for 3 days were detected by FACS using Annexin V‐FITC/PI double staining. A representative experiment (n=3) is shown. [file MOL2-14-2701-s001.pdf]
