# Supplementary material for: Rho‐associated protein kinase‐dependent moesin phosphorylation is required for PD‐L1 stabilization in breast cancer
Source: Mol Oncol. 2020 Oct 3;14(11):2701–12. doi: 10.1002/1878-0261.12804 (PMC7607174; doi:10.1002/1878-0261.12804)

CBTCCCA

Certificate of STR Analysis

1.Sample

MDA-MB-231

2.2.Methods

The genomic DNA was purified with Purelink@ Genomic DNA Kits in our Bank.

The DNA sample was analysed in Ministry of public security Evidence Identification Center.

The sample was amplified with AmpFlSTR® Identifiler® PCR Amplification Kit.

The profiles STR loci and Amelogenin gene were characterized on ABI 3100 Type Genetic Analysis Instrument.

3.Results

D5S818	12
D13S317	13
D7S820	8,9
D16S539	12
vWA	15,18
TH01	7,9.3
Amelogenin	X
TPOX	8,9
CSF1PO	12,13

The above results were consistent with the DNA profiles reported by ATCC and DSMZ, and indicated no other human cell lines contamination.

中国科学院典型培养物保藏委员会
细胞库

Cell Bank,
Type Culture Collection,
Chinese Academy of Sciences
(CBTCCCAS)

2012/12/3

GeneMapper 4.0

Cell Line Authentication-8

Sample File	Sample Name	Panel	OS	SQ
61_E08_CellLineAuthentication-1-1229.fsa	BT549	STR Profile 1-human		

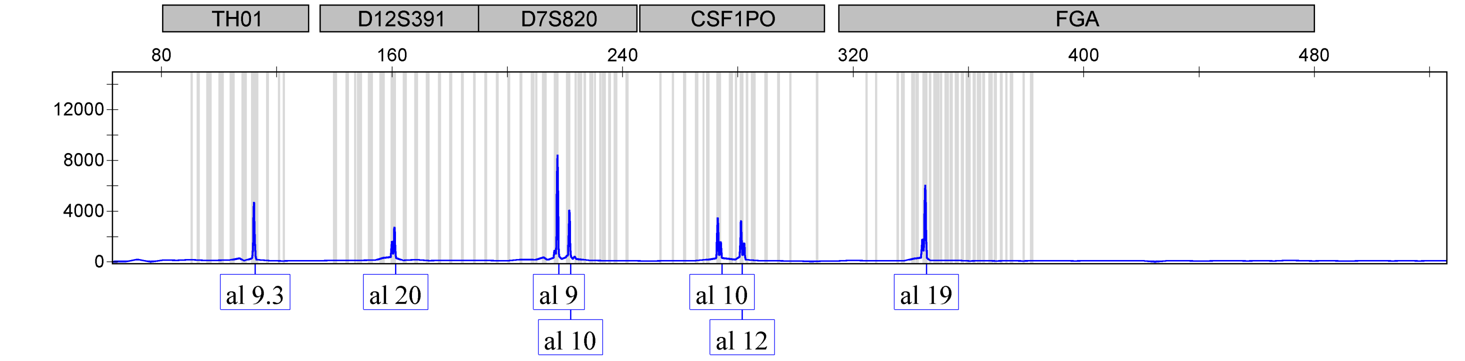

Sample File	Sample Name	Panel	OS	SQ
61_E08_CellLineAuthentication-1-1229.fsa	BT549	STR Profile 1-human		

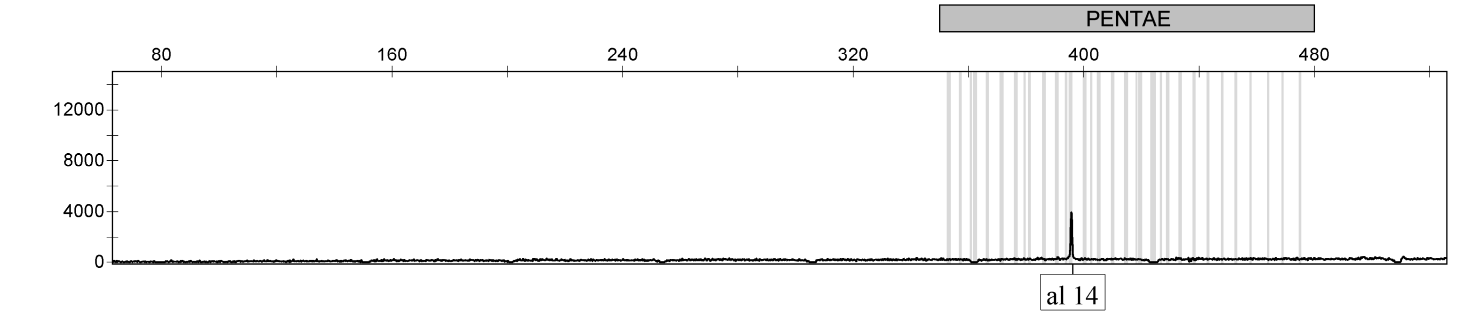

Sample File	Sample Name	Panel	OS	SQ
61_E08_CellLineAuthentication-3-1229.fsa	BT549	STR Profile 2-human		

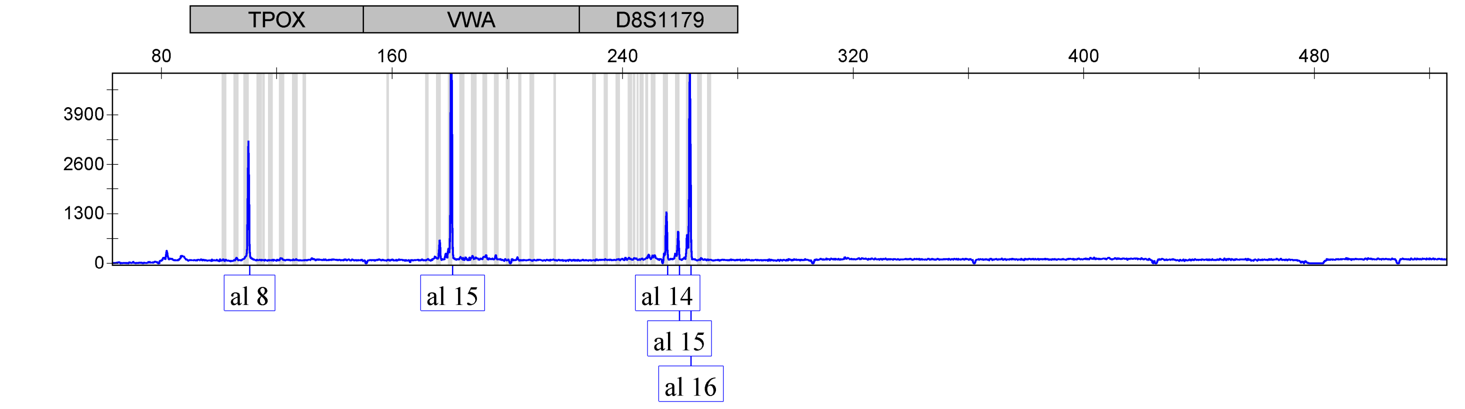

Applied Biosystems

GeneMapper 4.0

Cell Line Authentication-8

Sample File	Sample Name	Panel	OS	SQ
61 E08 CellLineAuthentication-3-1229.fsa	BT549	STR Profile 2-human		

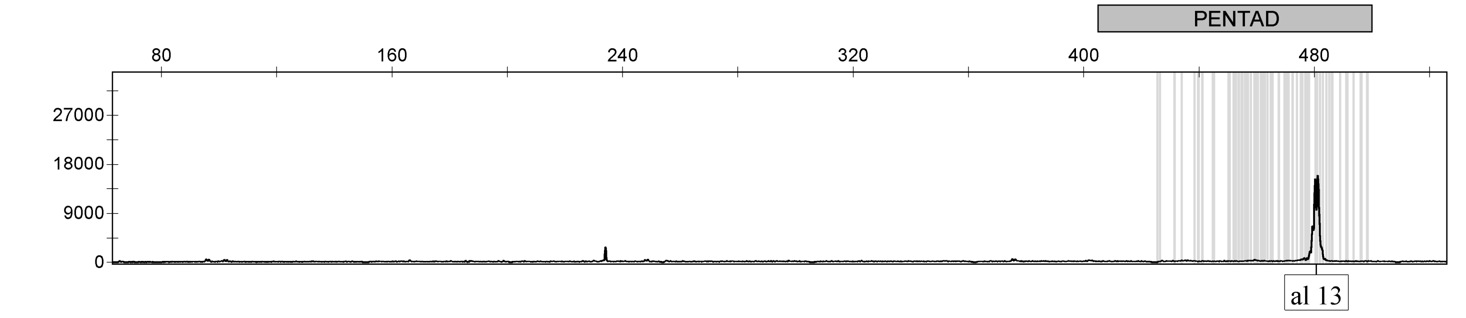

61 E08 CellLineAuthentication-4-1229.FSA	BT549	STR Profile 3-human		

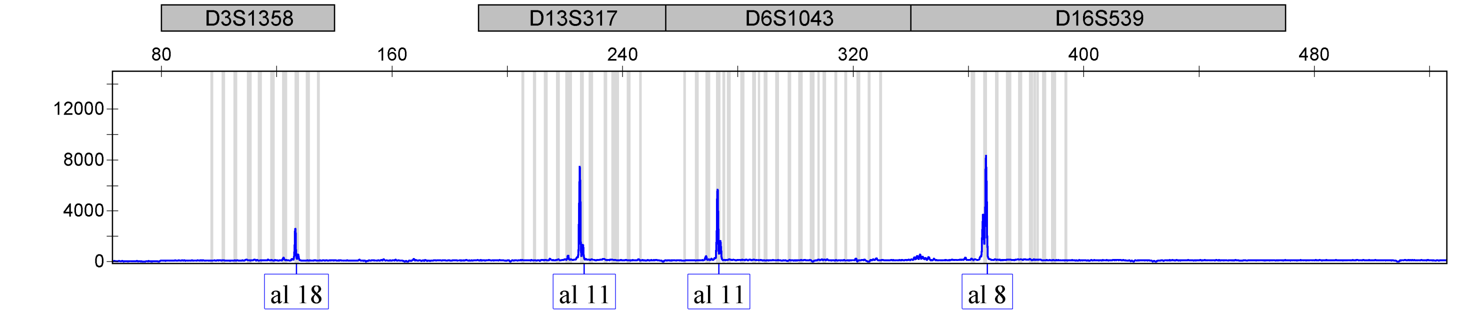

61 E08 CellLineAuthentication-4-1229.FSA	BT549	STR Profile 3-human		

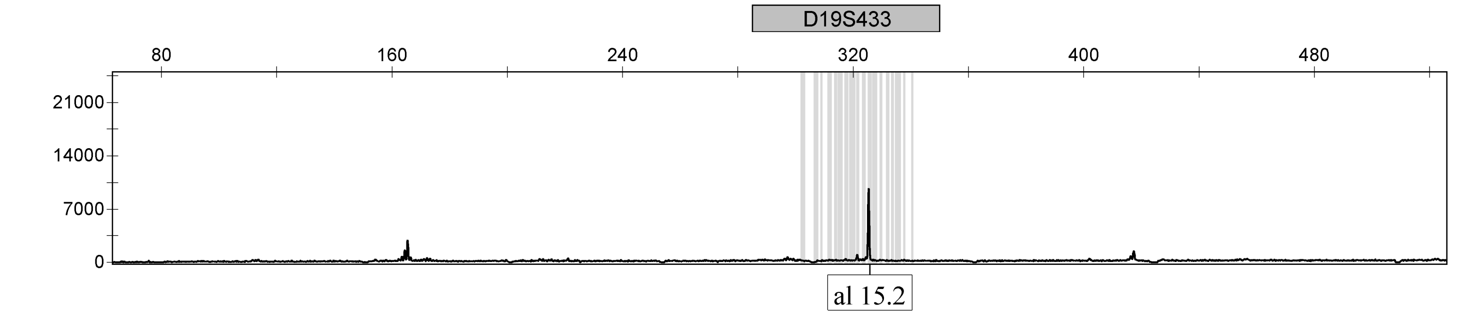

93 E12 CellLineAuthentication-3-1229.fsa	BT549	STR Profile 4-human		

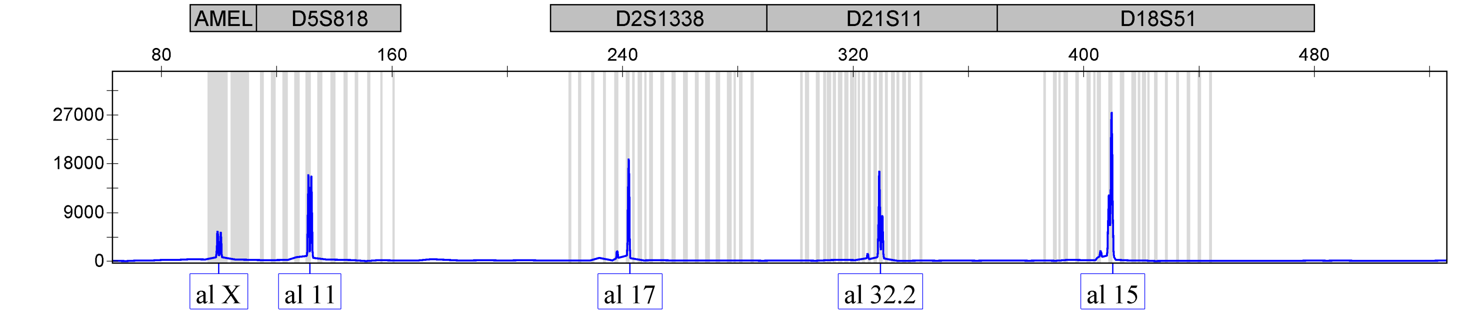

Sample File	Sample Name	Panel	OS	SQ
93_E12_CellLineAuthentication-3-1229.fsa	BT549	STR Profile 4-human		

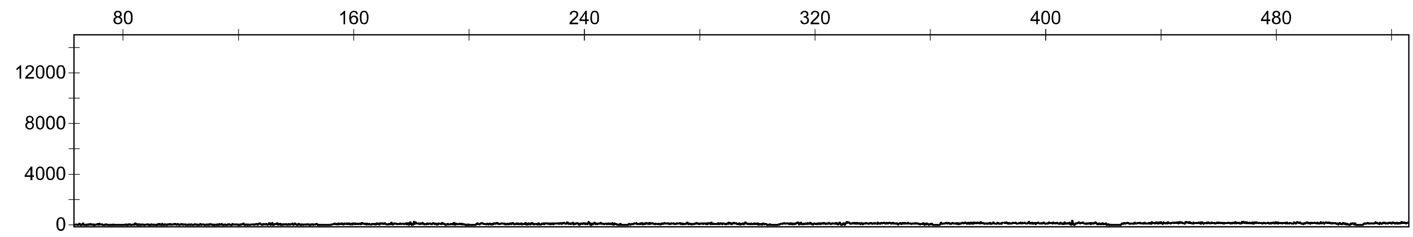

Supplement: Supplementary file 2 — Appendix S1 [file MOL2-14-2701-s002.pdf]
